# A microCT-based platform to quantify drug targeting

**DOI:** 10.1186/s41747-023-00355-8

**Published:** 2023-08-03

**Authors:** Brandon J. Ausk, Adam N. Tucker, Philippe Huber, Reza Firoozabadi, Jeffrey M. Gross, Ted S. Gross, Steven D. Bain

**Affiliations:** 1 In Situ Therapeutic Solutions Inc, Seattle, USA; 2 Department of Orthopaedics and Sports Medicine, University of Washington, Seattle, USA

**Keywords:** Drug delivery systems, Orthopedics, Ossification (heterotopic), Rabbits, X-ray microtomography

## Abstract

**Background:**

Heterotopic ossification (HO) is a frequent and debilitating complication of traumatic musculoskeletal injuries and orthopedic procedures. Prophylactic dosing of botulinum toxin type A (BTxA) holds potential as a novel treatment option if accurately distributed throughout soft-tissue volumes where protection is clinically desired. We developed a high-resolution, microcomputed tomography (microCT)-based imaging strategy to assess drug distribution and validated this platform by quantifying distribution achieved via a prototype delivery system *versus* a single-bolus injection.

**Methods:**

We injected an iodine-containing contrast agent (iodixanol 320 mg I/mL) into dissected rabbit musculature followed by microCT imaging and analysis. To contrast the performance of distributed *versus* bolus injections, a three-dimensional (3D) 64-cm^3^-printed soft-tissue holder was developed. A centered 2-cm^3^ volume of interest (VOI) was targeted with a single-bolus injection or an equal volume distributed injection delivered via a 3D-printed prototype. VOI drug coverage was quantified as a percentage of the VOI volume that was < 1.0 mm from the injected fluid.

**Results:**

The microCT-based approach enabled high-resolution quantification of injection distribution within soft tissue. The distributed dosing prototype provided significantly greater tissue coverage of the targeted VOI (72 ± 3%, mean ± standard deviation) when compared to an equal volume bolus dose (43 ± 5%, *p* = 0.031) while also enhancing the precision of injection targeting.

**Conclusions:**

A microCT-based imaging technique precisely quantifies drug distribution within a soft-tissue VOI, providing a path to overcome a barrier for clinical translation of prophylactic inhibition of HO by BTxA.

**Relevance statement:**

This platform will facilitate rapid optimization of injection parameters for clinical devices used to effectively and safely inhibit the formation of heterotopic ossification.

**Key points:**

• MicroCT provides high-resolution quantification of soft-tissue drug distribution.

• Distributed dosing is required to maximize soft-tissue drug coverage.

• Imaging platform will enable rapid screening of 3D-printed drug distribution prototypes.

**Graphical Abstract:**

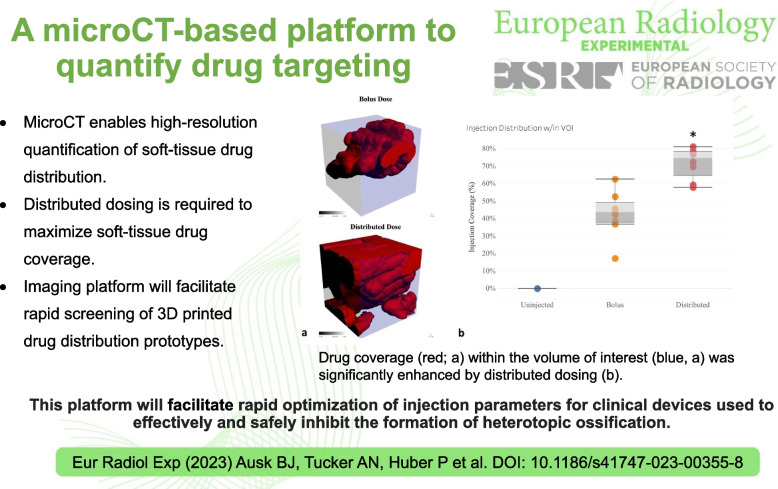

## Background

Heterotopic ossification (HO) is defined as bone formation that occurs in soft tissue outside the skeleton and is a frequent complication of surgical procedures required to address musculoskeletal traumas [1–3]. As the location of HO and trauma is commonly coincident, HO frequently forms near joints and causes pain, impaired movement, and, in the most severe cases, joint encapsulation leading to total loss of function [4, 5]. Once HO is symptomatic, the only treatment option is surgical excision of the ectopic bone, which is costly, technically demanding, time-consuming, and has high recurrence rates reported in the range of 6 to 36% predicated on anatomical location of the initial surgical procedure as well as surgical approach and timing [6–11].

The current standard of care for HO prevention is either high-dose-focused radiation therapy or a 1- to 6-week postoperative course of a nonsteroidal anti-inflammatory drugs (NSAID) such as indomethacin [12, 13]. The use of radiation therapy is limited by the lack of widespread availability, cost, and potential long-term complications [14–16]. The reported benefits of NSAID therapy vary considerably across studies and indications (*e.g.,* less effective for acetabular fracture fixation *versus* total hip replacement [17–20]). Recent studies have also raised concerns regarding the risk-to-reward ratio of NSAIDs as an HO treatment [21] due to elevated gastrointestinal bleeding, increased cardiovascular events, and impaired fracture healing [22–25]. We therefore believe that a prophylactic HO intervention via local treatment of tissue with demonstrably improved efficacy and safety *versus* NSAIDs will improve patient care and surgical outcomes.

Prophylactic treatment of soft tissue with botulinum toxin type A (BTxA) holds potential to achieve this goal. This strategy is derived from observations that trauma-induced disruption of neuromuscular signaling causes a neuroinflammatory cascade that, in turn, drives differentiation of pluripotent cells into the bone-forming osteoblasts that lead to HO [26–29]. In context with our recent studies of how neuromuscular dysfunction mediates bone cell activity [30–32], we speculated that BTxA-induced inhibition of neuromuscular function would inhibit HO. Using a murine model of bone morphogenetic protein-induced HO, we found that a single dose of BTxA reduced HO volume by 50% [33]. A series of subsequent studies then demonstrated that the effectiveness of this intervention was not due to the paralytic effect of BTxA but was spatially associated with the site where BTxA was injected [34]. Thus, translation of this intervention will require a device that enables targeted delivery of distributed small doses of BTxA within the muscle volume where protection from HO-related pain and dysfunction is clinically desirable.

Unfortunately, this translational goal is not currently achievable as previous imaging strategies to visualize distribution of injected fluid within soft tissue (*e.g.*, magnetic resonance imaging) have a native resolution nearly an order of magnitude lower than that is needed for this purpose [35]. In this study, we address this technical barrier by developing a microcomputed tomography (microCT)-based platform to quantify three-dimensional (3D) drug distribution within muscle. To validate our approach, we assessed whether a prototype 3D-printed distribution device enhanced drug distribution within a target volume of interest *versus* a bolus dose.

## Methods

### Study design overview

All studies were performed *ex vivo* with either harvested (leporine) or procured (porcine) tissues. Quantification of injection distribution was achieved through a combination of contrast agent injection and microCT scanning. A digital microinjection system was used to maximize reproducibility of injection volumes and control injection conditions, while a custom 3D-printed injection template was used to guide and deliver the contrast agent to tissue volumes of interest (VOIs). To first determine if the imaging strategy applied could evaluate injection distribution in our soft-tissue model, we quantified precise imaging thresholds required for microCT visualization of the injected contrast agent. We next applied this imaging strategy to quantify contrast agent in the VOI following either a distributed dosing protocol *versus* a single-bolus dose and compared these two approaches for their respective ability to provide drug coverage in the VOI.

### Injection and microCT scanning

**Fig. 1:**
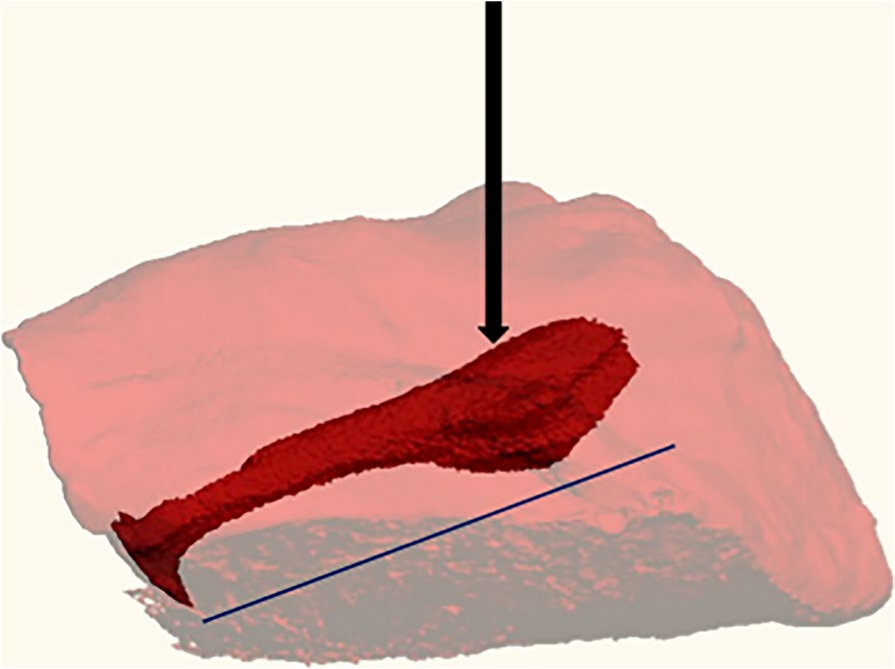
MicroCT rendering of a single injection. A 5.6-cm^3^ abductor muscle volume (light red) with a single 60-µL injection (arrow) oblique to the primary muscle fiber orientation of the sample (angling from upper right to lower left; blue line). The distributed volume was a three-dimensional spindle aligned with the muscle fiber orientation (dark red)

All injections were performed using a UMP3 UltraMicroPump coupled with a Micro4 controller (World Precision Instruments, Sarasota, USA) which aliquoted precise injection volumes at a rate of 10 µL/s. A 500-µL syringe (Trajan Scientific and Medical, Victoria, Australia) coupled with a 2″ 25-G beveled needle (Trajan) was used in all studies. Iodixanol, an iodinated x-ray contrast agent generally used for angiography, was chosen for microCT injection visualization. We used a 33% iodixanol 320 mg I/mL (Visipaque^®^, General Electric Healthcare, Chicago, USA) dilution (2:1 injectable saline to Visipaque 320; viscosity 5.5 cP) as a model injectable solution. All specimens were imaged using a vivaCT 40 scanner (Scanco Medical, Wanger-Brüttisellen, Switzerland) (voxel resolution 38 µm, 55 kVp, 145 µA, 200 ms). To provide an initial proof of concept for this technique, a portion of rabbit abductor muscle (5.6 cm^3^) was dissected from a freshly sacrificed rabbit, and a 60-µL bolus was injected. The specimen was imaged within 20 min and the distribution of the injection quantified by standard thresholding algorithms [36] (Fig. 1).

### Specimen holder and prototype injection template

**Fig. 2:**
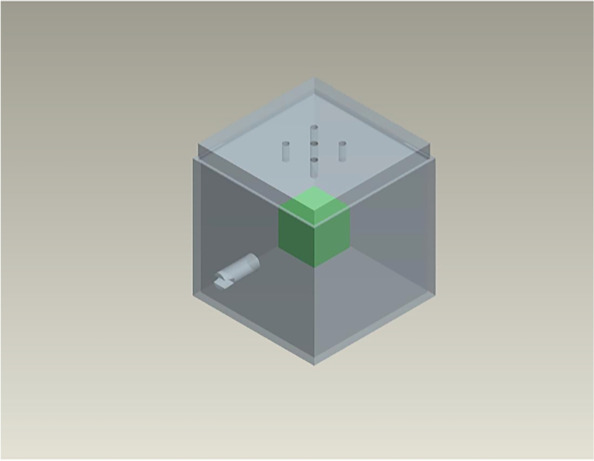
Three-dimensional printed injection specimen holder. The injection specimen holder (gray, volume 64 cm^3^) enabled quantification of drug distribution strategies within a targeted volume of interest that was 3.1% of the total tissue volume (green, volume 2 cm^3^). Drug patterning was achieved by altering the injection port(s) orientation on the lid in combination with spacers that precisely controlled injection depth(s). The distributed dosing injection port pattern is visible on the lid

Injection distribution was quantified in porcine shoulder specimens. To minimize tissue handling postinjection, we developed a 3D-printed 4 × 4 × 4 cm^3^ inner dimension muscle specimen holder (64 cm^3^). The specimen holder was printed to enable capping with injection templates of various configurations and included a mounting attachment for microCT imaging (Fig. 2). For this study, we modeled a target VOI as a cubic box with a volume of 2 cm^3^ centered within the overall tissue volume (*i.e.*, the VOI was 3.1% of total tissue volume). Precision injections were achieved using a custom template (forming the lid of the specimen holder) that provided 5 spaced locations in a quincunx pattern and a series of 3-printed spacers for each injection depth for a total of 15 injection sites. All 5 injections were performed consecutively at a single depth before moving to the next depth (moving superior to inferior within the specimen). The bolus injection was made using the centered port and middle depth.

Tissue specimens were randomly assigned to one of three experimental groups (*n* = 8 per group): group 1 – a single-bolus injection targeted to the center of the VOI (bolus, total injection volume 450 µL); group 2 consisting of 15 injections, each 30-μL distributed throughout the VOI (Distributed); and group 3 naive controls (Uninjected). Immediately following completion of assigned injections, the entire test specimen holder was imaged with tissue specimen in place.

### Quantification of fluid distribution in muscle

All high-resolution microCT images were acquired using a Scanco vivaCT 40. Specifically, a 38-µm voxel resolution scan was obtained of the center of the injection specimen holder to quantify both the VOI and a sufficient volume of surrounding tissue (cylindrical scan volume; diameter 3.7 cm, length 2.0 cm). To remove image noise and enhance the edge detection of injection volumes, all raw images were passed through a Gaussian filter (sigma 1.2, support 2.0), followed by identification of the targeted VOI in each scan.

**Fig. 3:**
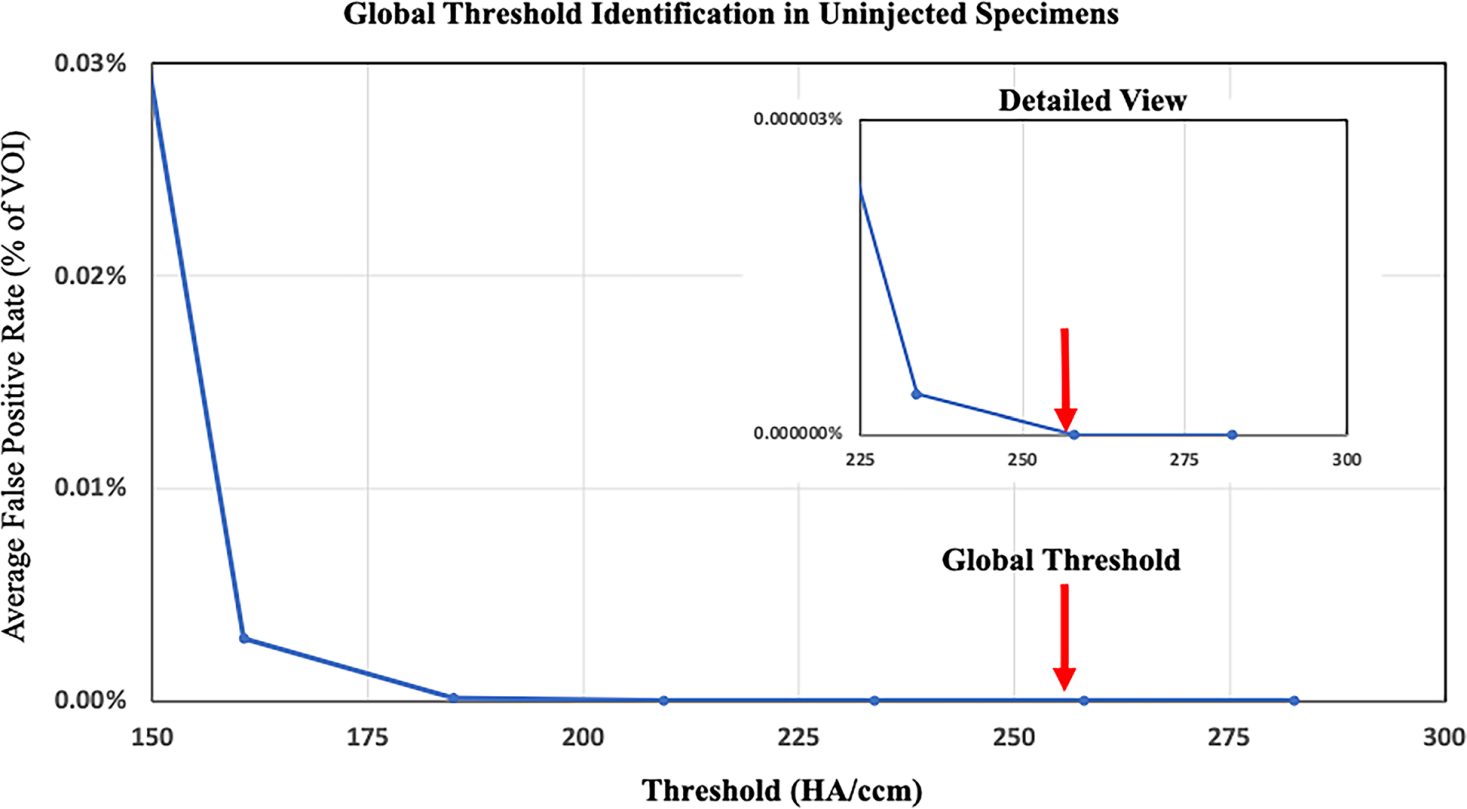
MicroCT threshold determination. Following image smoothing with a Gaussian filter, the volumes of interest of Uninjected specimens were segmented across a range of threshold levels to determine the lowest threshold capable of separating soft-tissue and injection of the iodine contrast agent. The global threshold of 258 hydroxyapatite (HA)/cm^3^ (red arrow) was chosen as it produced no false positives (*i.e.*, soft tissue incorrectly identified as injected with contrast agent) and applied throughout the study

**Fig. 4:**
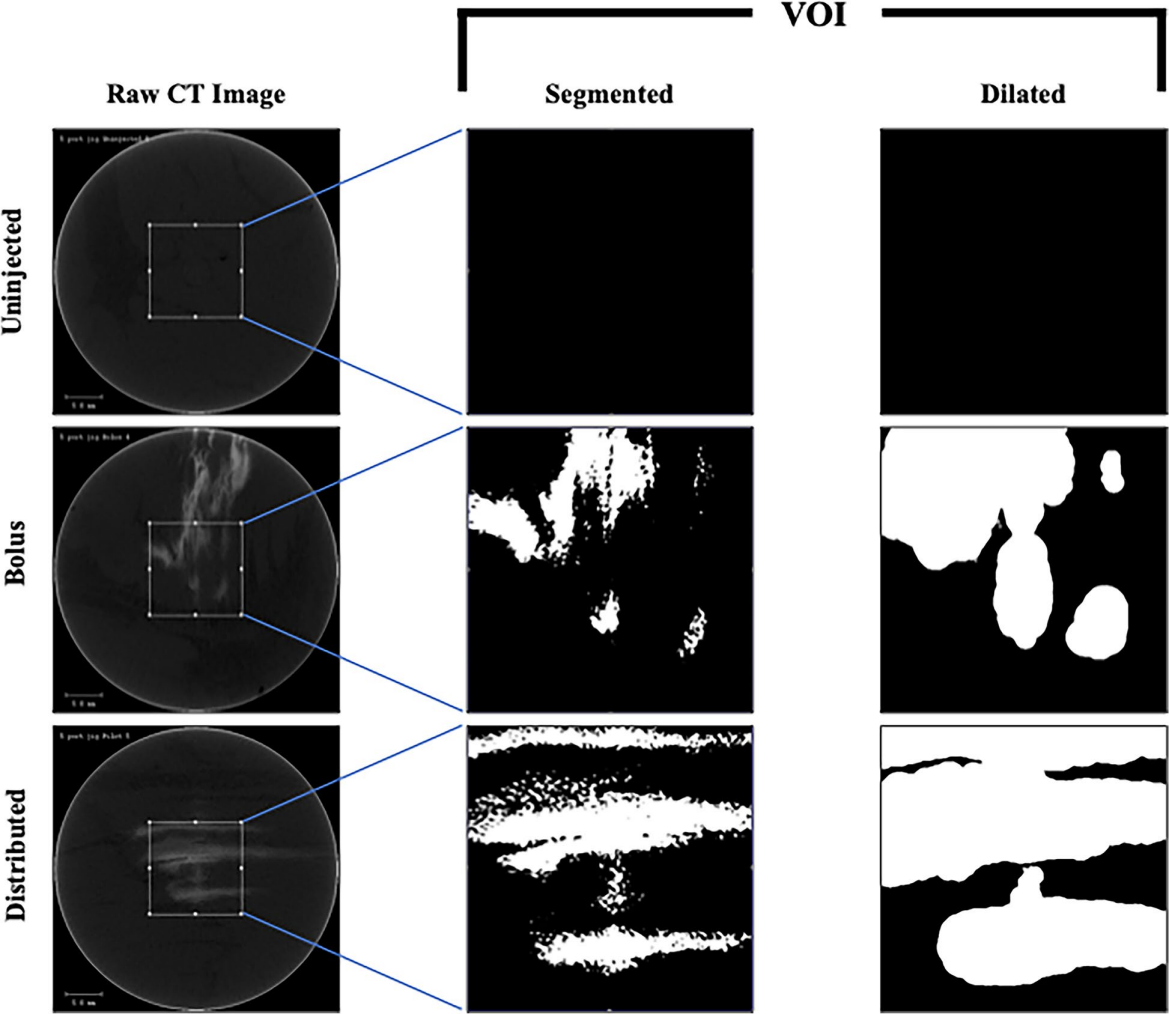
Image processing algorithm from image acquisition to quantification. Representative raw two-dimensional microCT images from Uninjected, bolus, and distributed dosing groups. The two-dimensional images are from the center of the volume of interest (VOI, white box). Standard microCT filtering and thresholding were applied to quantify drug distribution within each VOI (segmented). Finally, the drug coverage was quantified by three-dimensional dilation of the segmented and filtered image (dilated, < 1 mm)

To determine the imaging threshold required to visualize the contrast agent, we performed a parametric examination of the Uninjected group. From this analysis, a threshold of 258 hydroxyapatite (HA)/cm^3^ was identified as the minimum required to produce zero false-positive voxels in the Uninjected group (Fig. 3). This threshold was then applied to all samples to identify injection distribution within the targeted VOI (Fig. 4). Per standard image thresholding techniques, a visual inspection of the correspondence between two-dimensional binarized images and the original grayscale images was performed to confirm the validity of the threshold value chosen (Fig. 4). Given our previous experimental data suggesting that soft tissue within 1 mm of BTxA injection would be protected from HO formation [34], we next performed a 3D dilation on the segmented volumes (MATLAB, Fig. 4). This dilated volume was used to quantify our primary outcome measure, the effective drug coverage (*i.e.*, the percentage of the targeted VOI that was within 1 mm of any voxel determined to contain injected fluid). As a secondary measure, we quantified off-target injected drug (*i.e.*, dosing that is likely ineffective for this targeted application). We quantified injected fluid outside of concentric volumes larger than the VOI in 1 cm^3^ increments from 4 to 7 cm^3^ as a percentage of total injected fluid volume.

### Statistics

Two outcome measures were quantified: (1) effective drug coverage within the targeted VOI and (2) off-target fluid volume. One-way ANOVA were used to determine differences in effective drug coverage between Bolus, Distributed, and Uninjected groups. As homogeneity of variance was not observed between the groups, a nonparametric Kruskal-Wallis test with a post hoc Dunn test was performed. Similarly, differences in off-target fluid volumes in Bolus and Distributed dosing groups were determined by pairwise comparison using Mann-Whitney *U*-tests. The *p*-values lower than 0.05 were considered significant.

## Results

**Fig. 5:**
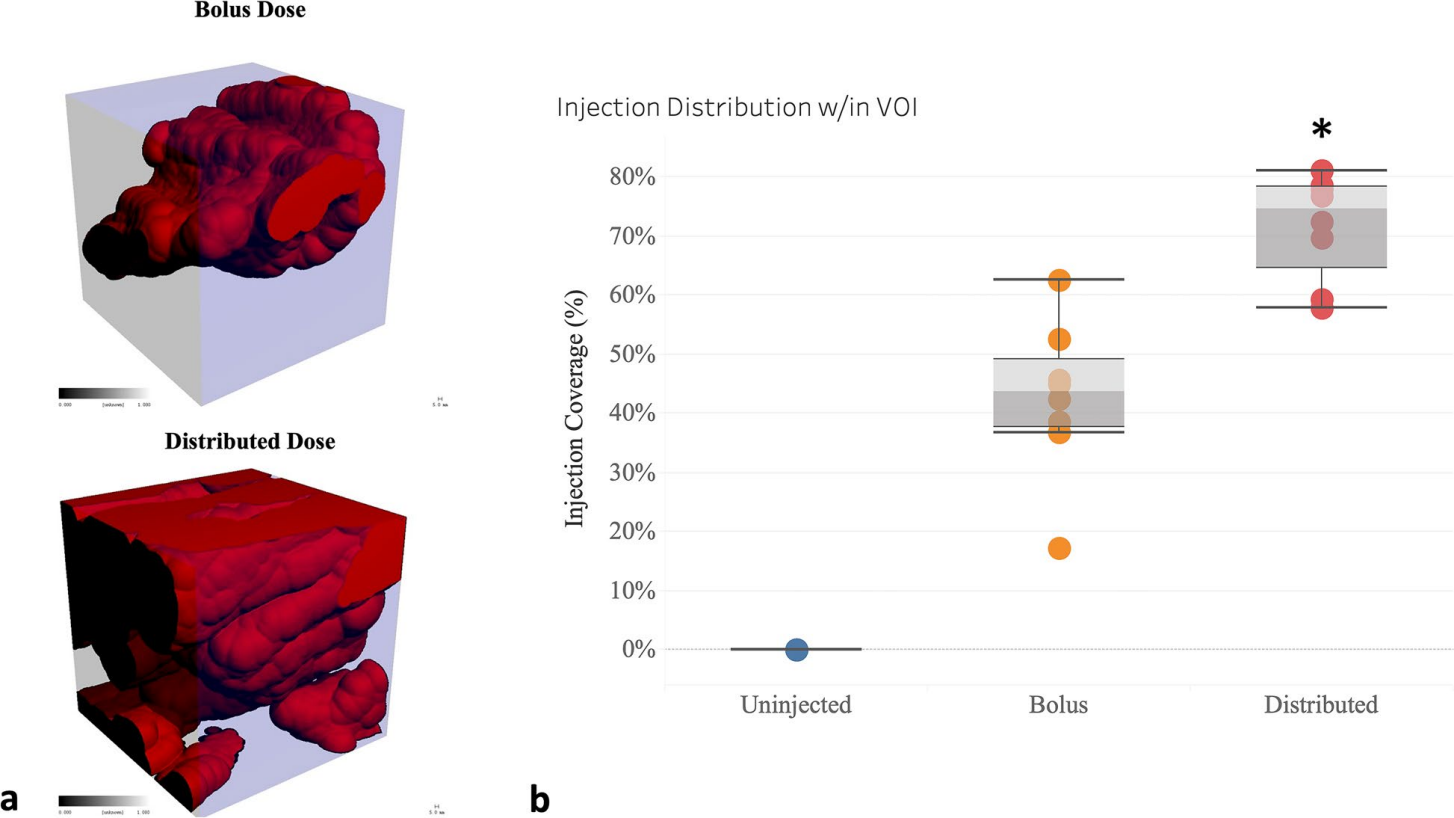
Bolus *versus* distributed drug coverage. Drug coverage (red) within the volume of interest (VOI, blue box) was significantly enhanced by distributed dosing (**a**). To visualize the drug distribution within the VOI, the dilation is constrained by the boundaries of the VOI. Box and whisker plot (**b**) illustrating the drug coverage within the target VOI. Distributed dosing increased median coverage by 70% when compared to bolus dosing (*p = 0.031)

**Fig. 6:**
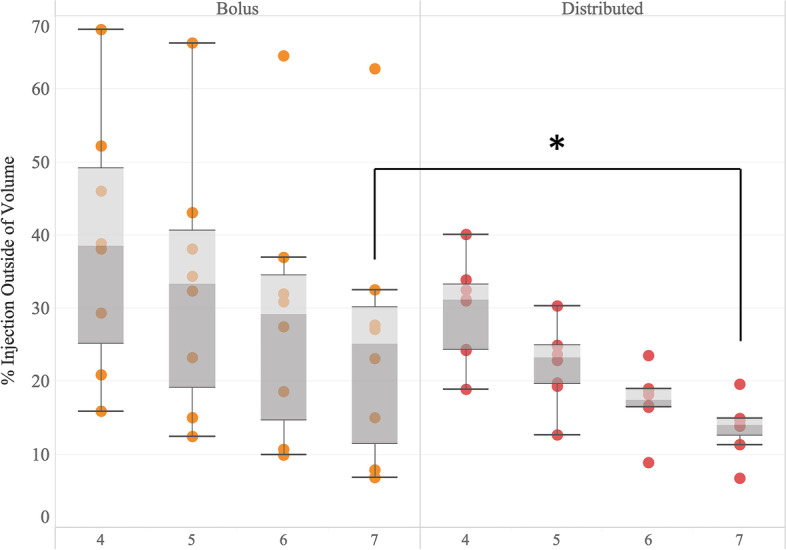
Off-target fluid volume. Box and whisker plots illustrating the percentage of injection outside of increasing concentric volumes centered about the 2-cm^3^ volume of interest (from 4 to 7 cm^3^). Bolus injections (orange) demonstrated elevated variability *versus* distributed dosing (red). As volume was increased, median escaped fluid volume was reduced by distributed dosing, reaching significance outside of 7 cm^3^ (*p = 0.041)

Injection coverage within the targeted VOI was significantly increased in the Distributed group compared with the Bolus group (Fig. 5). Specifically, a bolus injection targeted at the center of the VOI produced a median injection drug coverage of 44% of the VOI (mean 43%, Fig. 5, bolus). Distributing the same injection volume via the prototype delivery template significantly increased the median injection coverage (median 75%, mean 72%, *p* = 0.031, Fig. 5, Distributed). Finally, bolus dose off-target fluid volume exceeded that of distributed dosing outside of a 7 cm^3^ region centered on the VOI (+86%, *p* = 0.041, Fig. 6).

## Discussion

The inability to visualize and quantify soft-tissue injection volumes at high resolution impairs the ability to rigorously explore how injection parameters (*e.g.*, needle spacing, injection conditions) alter the precision and accuracy of drug distribution within complex muscular anatomy. We developed a microCT-based platform that overcomes this barrier. We assessed the feasibility of this approach by comparing on- and off-target fluid distribution for equal volume bolus *versus* distributed injections within a tissue volume that was 30 times larger than the targeted VOI. We observed that the 3D-printed prototype drug distribution device enhanced drug coverage within the VOI and reduced off-target injected fluid volume.

Given our interest in developing a platform that would be scalable at various stages of clinical translation (*i.e.*, to large animal model and human cadaver testing), we created a 3D-printed radiolucent injection specimen holder that would enable rapid parametric assessment of injection distribution prototypes. We then used an automated threshold identification protocol to define the diffuse edges of the injection and developed postprocessing algorithms to quantify effective fluid coverage within the VOI and off-target injected fluid outside the VOI. The combination of microCT and an iodinated contrast agent enabled high-resolution visualizations of the injection fields. This was anticipated as similar approaches have been used in varied contexts, albeit with much reduced (3 × to 15 ×) image resolution [37, 38]. For example, visualization of bolus insulin injection in the subcutaneous fat layer of anesthetized pigs successfully quantified injection distribution variation in nonstriated tissue [37]. CT has also been used to produce intramuscular fiducial markers for tumor bed identification [38]. Unlike our study, the authors created a “sticky” injectable that minimally diffused in order to image tumor resection surfaces. In the context of these studies, our data provides further support for the potential of this approach to successfully image fluids of varied viscosity.

Our study has several limitations. First, we precisely quantified drug distribution in *ex vivo* specimens. Our goal is to use this technique to optimize the design of devices to inhibit HO in live animal and clinical applications. We recognize that the final distribution of injections will vary *in vivo* due to factors that alter drug profusion/absorption such as muscle disruption (by trauma or surgery) and muscle contraction. However, we believe that the insight gained through *ex vivo* studies (excised rabbit tissue for preclinical testing and cadaveric samples for clinical applications) will enable development of injection devices whose soft-tissue coverage profile will significantly exceed freehand injection. Ultimately, the proof of this strategy will occur not by imaging injection distribution in live tissue but also by correlating HO inhibition in the soft-tissue volumes we target for protection. Another limitation is that we quantified on-target and off-target injected fluid differently. We defined effective “drug” coverage within the VOI as on-target, as an optimal intervention will most effectively distribute the least required drug to achieve efficacy. We implemented a 1-mm dilation around any voxel identified as fluid based on preclinical preliminary studies confirming that the beneficial effect of the intervention was mitigated 4 mm away from the injected site [34]. While altering this parameter would alter absolute values, it is clear from visual image inspection that our platform readily identifies varied fluid distributions within a small VOI (Fig. 4). Off-target dosing (*i.e.*, dosing that would not effectively inhibit HO in the targeted region) was reduced by the distributed dosing strategy. Although the relative difference between experimental conditions may have been influenced by our confined tissue test volume, we believe that the centered bolus dose provides a conservative comparison for the efficacy of distributed dosing. Finally, we used a single viscosity solution similar to blood (3.5–5.5 cP, [39]), which is higher than the injectable solution being modeled (BTxA in saline). We would anticipate that altering injection viscosity would alter absolute, but not relative results. Given the ability of our platform to image fluid of varied viscosity, future parametric studies will be conducted to confirm this thesis.

Our approach focused on developing a platform and outcome measures capable of detecting differences in injected fluid distribution at sufficient resolution to enable future optimization. We implemented a best-case bolus condition as the bolus injection was centered within the VOI (*i.e.*, 0.63 cm from outer edge of the VOI). In contrast, 80% of the distributed prototype injections (12 of 15) were within 0.13 cm of an outer edge of the VOI. Our goal was not to optimize a distribution strategy, as we recognize that many variables of a drug delivery system must to be explored to achieve this objective (*e.g.*, injection volume, injection speed, number of injections, spacing). However, these data emphasize that a distribution device is essential, as even an unoptimized prototype significantly improved effective drug coverage and reduced off-target injected fluid. Practically, this is a manifestation of a portion of the injected fluid migrating along muscle fiber orientation away from the targeted VOI, a phenomenon that is likely to be exacerbated within a complex surgical field with multiple muscle fiber orientations and surgical disruptions. This injection phenomenon highlights the difficulty inherent in using BTxA as HO prophylaxis, as targeting a variety of endplate zones in muscle will require a diffuse distribution of drug [35, 40] while seeking the minimize effective dose for drug safety considerations. Ultimately, our data suggest that although freehand manual delivery (as approximated by the spatially centered bolus injection) may randomly be accurate, the elevated variability of this strategy is not desirable. Distributed dosing will not only enhanced targeting precision (thus minimizing the required dose of BTxA), but it will also reduce the potential for excessive and/or off-target BTxA to induce undesirable musculoskeletal side effects [41, 42].

In summary, we have developed a microCT-based platform capable of quantifying drug distribution within muscle at a 38-µm voxel resolution. As the injection locations and depths of a 3D-printed template can be readily permutated, this platform will enable rapid optimization of injection parameters (*e.g.*, port locations, dose volume, injection speed, and needle gauge) to most efficiently target a soft-tissue region where HO inhibition is desired while minimizing off-target injected fluid.

## Data Availability

All data generated or analyzed during this study are included in this published article.

## Abbreviations

3D: Three-dimensional
BTxA: Botulinum toxin type A
HO: Heterotopic ossification
MicroCT: Microcomputed tomography
NSAIDs: Nonsteroidal anti-inflammatory drugs
VOI: Volume of interest
